# Annotations

**Published:** 1894-08-18

**Authors:** 


### ANNOTATIONS.

### The Medical Confessional.

In many ways the great railway strike which recently caused so much anxiety in the United States was not conducted with much regard for that fair play which should rule in an industrial struggle in which there is, presumably, a desire for justice on both sides; but one incident stands out as a peculiar infraction of the laws of honour. There was published in all the newspapers a telegram, purporting to be from a New York doctor who had attended Eugene Debs, the leader of the strikers, for insanity due to drunkenness, and containing a warning that the excitement of his present action might lead to a return of the mania. The intention was evidently to disparage Debs in the eyes of the public, and shake his followers' confidence in his leadership. One would have liked to believe that the telegram was a mere forgery, but no repudiation of it ever appeared. If, as we must therefore infer, it was a genuine document, its publication was a direct violation of the confidence which should exist between doctor and patient. If the doctor thought Debs's intellect was in danger he might legitimately have conveyed a private warning to him or his friends; but to take a wide and largely hostile public into the secrets of a man's past is no part of a physician's duty. Information gained in the consulting room is as sacred as that given to the priest in the confessional, and advice based on it should be given to the patient's private ear alone, or, at need, to that of his nearest and most loyal friend. To advertise his past misfortunes or faults in the public prints is an act which is, we hope, as rare as it is unprofessional. Debs was neither mad nor drunk when the telegram appeared, but had he been both his credit could not have been more completely shattered than by this accusation couched as a warning. We have nothing to do, in these columns, with the wisdom or folly of the railway strike, nor with the motive that incited Debs to organise it; but it is within our province to protest, and that strongly, against the conduct of every doctor who is disloyal to his patient.

### The Lungs of London.

The *New Review* contains an excellent article by the Earl of Meath on the possibilities of metropolitan parks, which ought to be widely read, and prove fruitful in benefit to poorer Londoners. The suggestion is that during the summer months, and especially during August, it would be of great benefit to the people if they could be attracted and kept in the parks during the cooler hours of the evening. To ensure this result Lord Meath urges that steps should be taken to light the parks by electricity, in the same way that the London County Council has decided to light the Victoria Embankment Gardens and the bridges leading thereto. Lord Meath further urges the erection and maintenance of small, well-sheltered playgrounds, fitted up with gymnastic apparatus, and placed under the supervision of respectable and able-bodied women. But Londoners need amusement as well as air and exercise, and it would be well to promote evening concerts in the parks during the fine weather, like those which have proved so popular in Brussels and other Continental cities. The London County Council, to its credit, has set an example to the Government department which is responsible for the Royal enclosures in matters of this kind, which it would be well for it to emulate. What a boon it would be if Lord Meath's suggestion could be carried out to convert the Thames and its neighbouring embankments into a pleasure resort, where open-air or semi open-air entertainments of a popular nature took place throughout the summer! The climate of London and of Paris in summer time is not, after all, widely dissimilar, and as Lord Meath's suggestions are eminently practicable, we hope they may speedily become accomplished facts. Then the "lungs of London" would provide an additional boon to all classes of residents.

### Radcliffe Infirmary, Oxford.

We gather from the *Oxford Chronicle*, and the proceedings at the quarterly court of governors, that a reorganisation of the management department of the Radcliffe Infirmary is essential to its successful development. The governors, having determined to remodel the old buildings and to erect a new wing, and having completed the work with despatch, must now take the finances in hand. It would be too grave a reflection upon the Committee of Management, now that the new buildings have been erected, for them to consent to close the accident ward and a portion of the children's block, and to keep empty the old house which is henceforward to be devoted to the administration because a sufficient income is not forthcoming to maintain them. The crisis which the committee have to face at Oxford at the present time is identical with that which had to be faced by University College Hospital some fifteen years ago. We know nothing of the *personnel* of the management at Oxford, but the committee ought to recognise that it is impossible at the present day to finance a voluntary institution unless there is at the head of the executive staff a man of great energy and special training. We have not failed to urge upon committees the importance of securing trained men of this type for the post of secretary and superintendent. At Norwich, at Birmingham, and at Manchester, the reorganisation of the managerial staff and the appointment of a trained expert, at an adequate salary, to take charge of the financial and lay administration, has been attended with the greatest success. The time has arrived when, in our opinion, the authorities at the Radcliffe Infirmary should appoint a special committee, charged with the duty of modernising the management. If they do this they will find, no doubt, as other hospitals have found, that funds will flow in to an adequate extent, and that the necessity for closing any portion of the buildings, which the needs of the poorer population demand should be kept in active operation, would cease. Lord Dillon has shown himself an active and able treasurer, and we are confident that this suggestion will receive immediate attention with the view of making the Radcliffe Infirmary, under his administration, worthy of the best traditions of the noble University and ancient city of Oxford. Money cannot be procured for voluntary charities in these days unless the management adopts adequate methods to raise it. Close personal attention on the part of the permanent and paid chief of the executive staff is essential to success. Such a chief must possess experience, initiative, and whole-hearted devotion, combined with at least equal energy to that displayed by those who have to compete for a living as business men with the best brains which the commercial world can produce. There is nothing miraculous about financing a hospital. It simply requires the same attention and energy as any other business undertaking, and these have been sadly lacking, so far, in the conduct of affairs of the Radcliffe Infirmary. Remembering, as we do with appreciation, the courage and energy displayed by Dr. Bright in putting the buildings into order, we are confident that Lord Dillon will speedily show that the general finances can be improved to the requisite extent by a wise rearrangement of the lay administration.

